# The Eco-Agricultural Industrial Chain: The Meaning, Content and Practices

**DOI:** 10.3390/ijerph20043281

**Published:** 2023-02-13

**Authors:** Yongwei Liu, Zhenzhen Yang, Changxiong Zhu, Baogang Zhang, Hongna Li

**Affiliations:** 1 Key Laboratory of Groundwater Circulation and Evolution, Ministry of Education, School of Water Resources and Environment, China University of Geosciences Beijing, Beijing 100083, China; 2 Institute of Environment and Sustainable Development in Agriculture, Chinese Academy of Agricultural Sciences, Beijing 100081, China

**Keywords:** water pollution control, agricultural non-point pollution, eco-agricultural industrial chain

## Abstract

Lucid waters and lush mountains are invaluable assets. Resource-saving and environmentally friendly industrial structures, production, and living modes are pursued continuously for sustainable ecological development. According to the Second National Pollution-Source Survey, agricultural non-point pollution is still the most important source of the current water pollution. In order to improve the water environment and control the pollution, the meaning and content of the eco-agricultural industrial chain was introduced. Based on this conception, the eco-agricultural industrial chain, integrating a whole circular system with different sessions of crop farming, animal breeding, agricultural product processing, and rural living, was innovatively put forward to control the agricultural non-point pollution and protect the water environment systematically for the first time in this paper. The sustainable development was realized at a large scale from the reduction and harmlessness at the source, resource utilization in the process, and ecological restoration in the end. Core techniques were innovated based on the integration of agricultural industries to achieve the high-quality and green development of agriculture. The system included ecological breeding technologies, ecological cultivation technologies, as well as rural sewage treatment and recycling technologies, in the principle of reduce, reuse, and resource. Based on this, the agricultural production changed from the traditional mode of “resources–products–wastes” to the circulation pattern of “resources–products–renewable resources–products”. Thus, the final aim could be achieved to realize the material’s multilevel use and energy conversion in the system. The eco-agricultural industrial chain technology was proven to be efficient to achieve both the good control of agricultural non-point pollution and an effective improvement in the water quality.

## 1. Agricultural NPS Pollution

Agricultural non-point source (NPS) pollution mainly refers to contaminants from non-specific locations in agricultural production and rural lives, which cause air, soil, and water environment pollution in diffuse forms. Contrary to point source pollution, NPS pollution is heterogeneously distributed and characterized by random and intermittent occurrence, complex mechanisms and processes, uncertain discharge channels and amounts, and variable spatial and temporal pollution loads, and thus it is extremely difficult to control accurately by technical measures. Agricultural NPS pollution has aroused widespread concern throughout the world. It is now an important issue in water environment pollution, contributing much to water eutrophication in China as well as in other parts of the world [1]. In the United States, 80% of rivers and 85% of lakes are affected by non-point source pollution, which is the largest source of water pollution [2,3]. Agricultural activities contribute 28% of phosphorus, 70% of nitrate, and 76% of sediment to rivers in the United Kingdom [4]. Agricultural NPS is still the most important source of the current water pollution in China, according to the second National Pollution-Source Survey [5]. Available statistical data showed that industrial sources took a ratio of 2.50~5.12% in the total TN and TP entering the natural water, while agricultural NPS (45.52~67.23%) and domestic sewage (22.99~30.25%) contributed the vast majority [6]. A previous report indicated that the total farmland area polluted by agricultural NPS reached 2 million hm^2^, and almost half of the underground water was already contaminated [7]. The contribution rate of agricultural non-point source pollution is far more than that of point source pollution from industry and urban life, and has become the main pollution source of the most important river basins in China [8]. Agricultural NPS pollution has become the greatest problem in natural water, seriously affecting the sustainable development of agriculture and the environment [9]. Therefore, in order to fundamentally solve the problem of water pollution in China, we must place agricultural NPS pollution prevention and control into the important agenda of environmental protection [10,11].

The Environmental Protection Agency of United States announced in 2000 that agricultural NPS had become the main “contributor” to water pollution [12]. The pollution status of agricultural NPS was once severe in the European Union due to their high degree of agricultural modernization. Water pollution caused by nutrient loss from agricultural ecosystems was reported to be the primary source of pollution in surface water in EU countries [13,14]. For example, nitrogen accounts for 60–87% of the total pollutants in watersheds in Sweden, and 20% of lake water in Finland has already been polluted due to agricultural activities [15,16].

Agricultural NPS pollution is mainly caused by chemical fertilizer, pesticides, livestock and poultry manure, and other forms of agricultural waste. The sources are mainly pesticides and fertilizers lost due to irrational use in agricultural production, agricultural film left in cultivated land, improper disposal of animal manure, and pollutants produced by unscientific and irregular application. Specifically, the use of pesticides in China is ranked first in the world, with 0.73 million t in 1990, and a great increase to 1.16 million tons in 2017 [11]. In addition, the pesticides used were generally highly toxic and stable, with low utilization rates less than 30%, and the unused part was lost in the soil, water, and air [17]. The statistical data showed that the fertilizer amount in China increased from 41.24 million tons in 1999 to 54.04 million tons in 2019 [18,19]. The average application amount of fertilizer in China was up to 326 kg/hm^2^, more than two times the world average level [9]. The utilization rate of nitrogen (N), phosphorus (P), and potassium (K) had entered the international suitable range for the main food crops; however, the unused nutrients took a high ratio and were lost through runoff and leaching into the environment [20]. The contribution percentage of chemical fertilizer application to TN and TP reached 48.2% and 22.8%, respectively [11]. Livestock and poultry manure had a strong leaching ability and thus polluted water through surface runoff easily. It was estimated that the amount of animal manure increased by 136% during 1997–2018 [21]. According to the bulletin of the Second National Pollution Source Census of China in 2020, the total amount of chemical oxygen demand (COD), total nitrogen (TN), and total phosphorus (TP) of livestock and poultry breeding emissions reached 10.01, 0.60, and 0.12 million tons, respectively, in 2017. In aquaculture, fish manure, food sedimentation, and chemical fertilizers would also cause great pollution to the ecological environments of lakes and reservoirs [22]. At the same time, with the rapid growth of the population, improper or a lack treatment of rural wastewaters also resulted in a large degree of nitrogen and phosphorus pollution. Wu et al. found that rural living caused 38.1% of TP pollution in the Yanhe River watershed, which was the main source of its eutrophication [23]. In the Luanhe River Basin, rural living was the main factor causing TP pollution in agricultural NPS pollution, accounting 59.4% [24]. The production and accumulation of domestic sewage and municipal solid waste has aggravated the deterioration of the rural ecological environment, and it is one of the main sources of rural NPS pollution.

In general, the current situation of China’s agricultural NPS pollution is deteriorating, which has seriously affected the sustainable development of the environment and agriculture. Therefore, effective technology is extremely necessary to be developed for agricultural NPS pollution control.

## 2. The Eco-Agricultural Industrial Chain Technology

According to the Report of the Party’s 20th National Congress, green development and a harmonious coexistence between man and nature is constantly pursued in China. The eco-agricultural industrial chain technology is put forward according to the principle of reduce, reuse, and resource. It would guide agricultural production, changing from the traditional mode of “resources–products–wastes” to the circulation pattern of “resources–products–renewable resources–products”. Thus, the final aim could be achieved to realize the material’s multilevel use and energy conversion in the system. A number of anaerobic digestion (AD)-centered integrated systems have been reported in China, with various combinations of digester, animal production, and greenhouse [25]. In Liberia, an integrated system incorporating husbandry of pigs, rabbits, and fish with a rice mill was reported [25]. A” food to waste to food” system in Norway integrates AD, vegetable cultivation, and mushroom growing [26]. In this system, biogas production provides energy for the process and CO_2_ for the greenhouse, and, for the first time, the efficient direct use of digestate as a substrate and fertilizer was developed. The agricultural industry chain is suitable for the area incorporated with planting, breeding, and agricultural processing. In British Columbia, an Eco-Industrial Park was reported, including dairy farming, greenhouse cultivation of vegetables, and mushroom growing. The results indicated that non-renewable energy consumption, greenhouse gas emissions, aquatic acidification, respiratory effects from organic emissions, and human toxicity were reduced by 50%–90%. Meanwhile, aquatic eutrophication and respiratory effects from inorganic emissions also decreased by more than 10% [25]. Ecological ditches in the watersheds of modern agricultural parks could be used to intercept nitrogen and phosphorus from farmland runoff effectively [27]. It has been reported that ecological ditches can remove 24.9~72% of TN and 36.1~60% of TP [28]. A concatenation of the agricultural industry was chained to reduce costs and increase profits, and, more importantly, to control the agricultural NPS pollution and protect the water quality at the same time. In the United States, a series of policy systems and technical services have been developed to prevent and control the pollution of agricultural NPS. For example, the Food, Conservation, and Energy Act in 2008 required growers to comply with environmental conservation and wetland protection regulations, and the subsides and revenue insurance (including fallow fields) for crops are still provided in previous legislation. In addition, a series of supporting legal provisions, laws, and regulations have been introduced to guide famers to reduce the use of pesticides and other chemicals for the reduction of NPS at the same time. The enaction of the Soil and Water Quality Protection Act further sets out principal requirements for soil and water quality and protection. Moreover, the Department of Agriculture and the Environmental Protection Agency jointly launched and implemented an agricultural NPS pollution remediation program in the United States. In order to effectively control NPS pollution from various sources, the Clean Water Law has formulated different pollution emission standards based on the” Best Pollution Control Technology”, “Best Available Technology”, and “Best Practical Technology”. It also stipulates that the government will share a portion of the costs for those who voluntarily take measures to prevent and control agricultural NPS. Moreover, the government will grant tax deductions for those who voluntarily take other measures [12]. For the situation in Japan, they have proposed the concept of building a “Country of the Environment” that coexists with the Earth, and it has begun to emphasize the legalization of recycling and symbiosis in its environmental legislation and polices. In the case of rural wastewater treatment, for example, there are clear provisions in the relevant laws and regulations. In detail, for specific environmental preservation areas, sewerage treatment is jointly managed by the Ministry of Land, Infrastructure, and Transport; the Ministry of Agriculture, Forestry, and Fisheries; and the Ministry of the Environment. For agricultural promotion areas, agricultural village drainage facilities are used, and for other scattered rural areas, the technology of purification tanks is used [29]. Generally speaking, agricultural NPS management around the world has also undergone evolution from a local scale to an entire scale, from scattered points to an integrated system, and, finally, gradually toward collective joint development and utilization of the watershed as a unit.

The existing cases have fully proven the importance of the industrial chain integration in the control of agricultural NPS pollution and improvement of environmental quality. However, related work on the ecological agriculture industry chain is currently based on its operation law [30,31], stability evaluation [32], and dynamic mechanism [30]. The technical route needs to be further improved and integrated systematically. In addition, there is little research on eco-agricultural industrial chain technology in NPS pollution control, and relevant theoretical and engineering innovation practices are still lacking. Considering the serious situation of the water environment and the importance of agricultural NPS pollution control in China, there is an urgent need to carry out technological innovation and content improvement work on the eco-agricultural industrial chain technology.

## 3. The Eco-Agricultural Industrial Chain of “Crop Farming, Breeding, Agricultural Product Processing, and Rural Living”

The eco-agricultural industry chain is a circular integration system, merging independent chains of planting, breeding, processing of agricultural products, and rural life together. This paper, for the first time, puts forward the idea that the eco-agricultural industrial chain technology could be used to control the agricultural NPS pollution and to protect the water environment. The technical system focuses on source harmlessness, process resource utilization, end ecological treatment, and control in scale, and aims to achieve zero discharge of agricultural NPS pollutants and effectively improve the current situation of the water quality via joint control application. The key technologies involved include the following aspects.

### 3.1. Ecological Breeding Technologies

#### 3.1.1. Green Ecological Feed

With the development of intensive breeding and aquaculture, extensive hormone and antibiotic feed additives are used to lower the risk of diseases and increase production indexes, causing serious environment pollution [33]. Ecological feeds could maintain the microbial flora balance [34], enhance immunity [35], and promote digestion and nutrition absorption [36]. Moreover, they also could significantly accelerate animal growth, strengthen disease resistance, reduce the amount of feed, and improve the lean meat rate. For example, bioactive immunostimulants have been utilized to improve aquatic animal health, which act by boosting the immune systems of aquatic animals and improving the immunity levels against harmful microbes. They could improve growth, digestion, and immunity and modulate gut microbiota, and they are natural substances that are very friendly to the environment [35]. Microbial cell protein contains approximately 70% crude protein, the amino acid profile has been reported to resemble that of fish meal, and it has been produced by a new technology with upscaling and a low cost [36]. In addition, prebiotics, probiotics, essential oils, and organic acids were tested, with the aim to reduce or replace antimicrobials in feed [37]. Although ecological feed is relatively high-cost, and there is a lack of sufficient research on the formulation and processing technology at present, it would undoubtedly play a significant role in agricultural sustainable development and efficient source pollution control in breeding and aquaculture.

#### 3.1.2. Ecological Breeding Technology with Low Pollution and Zero Emissions

Ecological breeding is a sustainable development model of animal husbandry with low consumption, low emission, and high efficiency. The fermentation bed system (FBS) is an animal housing system of environmental protection, a safe and effective ecological pig raising mode combined with the modern microbial fermentation technology [38]. These systems mainly focus on reducing animal waste pollution, decreasing incidence, protecting animal welfare, improving animal product quality, and increasing breeding benefits, and can be used for recycling and sustainable production [39], as they are cheaper to establish compared to conventional confinement systems. This technology is based on the repeated spreading of sawdust, rice husk, corn stalks, or other agricultural material in indoor booths. A solid fermentation agent is added to build heap fermentation and then it is spread into the pig barn to form a bedding cushion. Animal feces and urine discharged directly in the bedding can be decomposed rapidly by padding materials, and the whole breeding process is zero-emission, with no smell and no pollution. It has been widely used in animal husbandry. Compared with traditional static composting, the continuous nitrogen addition of FBS could ensure that the bedding litters maintain appropriate C/N ratios for efficient microbial fermentation [40]. FBS is considered as an effective approach to deal with livestock manure, reducing ammonia nitrogen emissions and nitrogen losses in fermentation [40,41].

Han et al. proved that fermentation can prevent the presence of amines, in which beneficial microbes can decompose animal feces into fertilizer, eliminate odors, and inhibit the growth of pests and bacteria [42]. Zhou et al. studied the gaseous emissions, growth performance, and pork quality in a deep-litter system and concrete-floor system [36]. The results showed that piglets in the deep-litter system had some animal welfare improvements and odor nuisance reduction; moreover, pork quality was higher than that with the concrete-floor system [43]. In addition, other related studies showed that it was conducive to improving the sensory and edible quality for animal raising with FBS; meanwhile, FBS shows advantages in the reduction of NH_3_, CH_4_, N_2_O, and CO_2_ [44,45]. Different bedding materials could affect the fermentation process of animal manure in FBS, which would affect animals’ feeding environments, health, and production performance [39]. For example, corn stover has the greatest reduction in NH_3_ and wood chips reduce the emissions of CH_4_, N_2_O, and CO_2_ from animal feces [45]. FBS is a suitable pig raising mode with both economic benefits and environmental protection benefits, and thus it is worthy of popularization and application in the practice of agricultural non-point source pollution control.

#### 3.1.3. The Processing Technology of Biological Humic Acid Fertilizer with Padding Materials

Abandoned padding materials carry potential value as resources and are rich in humic acid, beneficial microbes, and nutrients (N + P_2_O_5_ + K_2_O ≥ 7%). These padding materials could be re-utilized as substrates for mushroom cultivation or organic fertilizers, with the principle of “adjusting measures to local conditions, and selecting methods to given quantity”. Dong et al. proved that after 55 days of composting of the padding materials, organic matter and total nutrient reached 6.19% and 56.11%, respectively, meeting the relevant regulations in the national agricultural industry standards for organic fertilizer. The abandoned padding materials of deep litter were thus evaluated as a safe source to produce integrated organic fertilizer [46]. Li et al. evaluated the fertilization of decomposed fillers, and found that the application of a filler increased the plant growth and soil nutrients. Moreover, it also mitigated soil acidification and salinization [47]. The nutrient loss of different fertilizers was tested under simulated rainfall conditions, and the results showed that, in the combined application of biological humic acid, runoff was reduced by 9.2%, 9.7%, and 17.5%, respectively, for chemical fertilizer, ordinary organic fertilizer, and biological organic fertilizer. TN loss was decreased by 27.8%, 42.2%, and 50.1%, and COD content was reduced by 36.6%, 20.7%, and 16.4%, respectively. These data indicated that the biological humic acid fertilizer produced from the raw materials of deep litter could effectively reduce the pollution of farmland runoff, showing not only rich nutritional value, but also considerable environmental benefits.

### 3.2. Ecological Crop Farming Technologies

#### 3.2.1. Agronomic Measures

Cover crops improve above- and below-ground biodiversity. The cover of a mixture of legumes or grasses increased the diversity of beneficial soil microbes while minimizing the proliferation of soil-borne pathogens [48]. Moreover, cover crops could control weeds, increase pest resistance, and reduce fertilizer costs [49]. Straw mulching could significantly reduce the surface runoff (73.9~86.2%), and showed more beneficial characteristics compared with plastic film mulching in minimizing the loss of nutrients and increasing the yield effect [50,51]. Compared with plastic film mulching, straw mulching showed greater potential for soil water storage before sowing under summer fallow mulching and year-round mulching. The nitrogen loss and greenhouse gas emissions of the year-round mulching were lower than those of summer fallow mulching. Therefore, straw mulching under year-round mulching should be recommended to local farmers in dryland areas of China and other similar areas around the world [50]. The rotation mode provides microbial richness and an increase in microbial diversity [52]. Well-planned crop rotation can reduce the infestation of fungi, bacteria, viruses, and insect pests, and control weed density [53]. Reduced tillage maintains soil health via the preservation of organic matter in the soil and minimal soil disturbance, which improves water regulation and reduces nutrient leaching into the groundwater, and leads to less soil erosion and improved carbon sequestration [49]. Intercropping could control pests, reduce N leaching, and increase soil N availability [49]. Intercropping includes mixed intercropping, relay intercropping, and strip intercropping, which incorporates a diversified plant community and thus increases above-ground biodiversity [49]. Compared with monocultures, intercropping reduced pest populations and N leaching, and increased soil N availability. Moreover, intercropping facilitates weed control through interspecific competition for light, nutrients, and water [54]. In addition, agroforestry [49], conservation agriculture [55], diversified crop–livestock systems [56], and organic agriculture [57] are good methods to control the farmland pollution from the source.

#### 3.2.2. Irrigation Modes

While many water-saving rice production techniques have been adopted in China, the environmental effects of these techniques require further investigation. Irrigated rice systems are the main rice ecosystems around the world. Several irrigation modes, including conventional flooding irrigation (CF), drip irrigation, semi-dry cultivation, shallow-wet irrigation (SW), controlled irrigation (CI), intermittent irrigation (II), no-flooded mulching cultivation, alternate wetting and drying irrigation (AWD), and rainfall-adapted irrigation (RAI), have been used to improve crop water productivity [58,59]. Among various irrigation modes, AWD has the most advantages in increasing famer incomes, reducing CH_4_ emissions, and alleviating pest attacks and disease damage [58,59]. RAI is a further development of AWD and has been widely practiced in multi-rain regions, which can not only maintain rice yields, but also reduce environmental pollution and the irrigation cost in comparison with CF [58,60]. Yan et al. investigated the effects of nitrogen management strategies on the grain yield, crop water productivity, and nitrogen use efficiency of rice under CF and RAI. The results showed that RAI enhanced the rainwater storage capacity and usage, also increased grain yields and nitrogen use efficiency, which increased grain yields and resource use efficiency by improving root growth in rice [58]. Water-saving irrigation has noticeable potential to alleviate water shortages and NPS pollution while ensuring high yields. Zhang et al. compared the effects of water saving, pollutant reducing, and yield increasing in four water-saving irrigation modes (SWI, CI, II, RAI) commonly used in China. The results showed that CI had the highest average water-saving rate and average pollutant-reducing rate, which were 35.12% and 54.97%, respectively, but the average yield-increasing rate was the lowest. In comparison, SWI was the most applicable water-saving irrigation mode and was suitable for 90.03% of paddy fields, followed by CI and II [59]. Drip irrigation is being used to ameliorate saline soil, and could supply water precisely and uniformly at high frequencies; meanwhile, it maintains high soil matric potential (SMP). Therefore, it is often used for the restoration of saline land [61,62]. Many studies have investigated drip irrigation for various crops in different saline soils in China [63,64,65,66]. Among them, drip irrigation has been explored in saline lands with arid and semi-arid climates in the Ningxia Plain, China [63], and was applied in saline wasteland with an inland arid climate in Xinjiang, Northwest China [65], as well as in saline-sodic soils in the Songnen Plain of China [64]. Drip irrigation technology brings economic benefits including water, fertilizer, and pesticide savings, as well as crop yield and income increases together.

#### 3.2.3. Fertilization System

Rationally controlling fertilizer application and strengthening fertilizer management can effectively reduce agricultural environmental pollution [67]. It is encouraged to apply the optimum formula for fertilization via soil testing to achieve a nutrient balance. The fertilization structure and application mode should be adjusted according to the local soil fertility for scientific farmland nutrient management. The use of slow-release fertilizer can reduce environmental pollution and improve nutrient utilization. At present, the most common slow-release fertilizers on the market mainly include coated slow-release fertilizers and chemical synthetic slow-release fertilizers [68]. Compared with coated controlled-release fertilizers, chemical synthetic slow-release fertilizers are more widely used because there is no film pollution [69]. Li et al. prepared a novel low-cost and high-efficiency slow-release fertilizer (PSRF/KCl). Compared with the traditionally chemical synthetic slow-release fertilizer, the raw material cost and energy consumption of PSRF/KCl were reduced by 17.51% and 38.13%, respectively, and it significantly improved the yield and quality of vegetables. The research gave chemical synthetic slow-release fertilizers the function of simultaneously releasing N, P, and K [70]. Moreover, fertilization methods also affect the use of water and fertilizer by crops. Compared with fertilization by broadcasting, shallow or deep band fertilization can improve the crop yield, and deep fertilizer placement is better [71]. Li et al. found that a nitrogen fertilizer deeply applied under the film-mulched band could reduce the ammonia volatilization loss and prevented nitrate leaching [72]. Side-deep fertilization could achieve the cleaner and light-simplified production of rice by reducing fertilizer use, improving fertilizer utilization, and alleviating labor shortages [73]. Studies have shown that in double-season rice areas, side-deep fertilization can synchronize the nitrogen supply with the plant demand, significantly reduce ammonia volatilization losses, improve nitrogen use efficiency, and increase rice yields based on reduced nitrogen application and frequency of nitrogen application [50,73]. These measures are all useful in nutrient loss control.

In conclusion, comprehensive factors including the local rainfall runoff, types of cultivated land, modes of farm planting, and the means of cultivation should be considered in the selection of ecological cultivation methods to control the nutrient losses from farmland planting.

### 3.3. Rural Sewage Treatment and Recycling Technology

Due to its large quantity, wide distribution, polluted area, intermittent discharge, and large coefficient of variation, rural domestic sewage has become a major cause of water quality deterioration [74]. Thus, the research and development of efficient decontamination treatments based on the local situation has become an important scientific subject. At present, the main treatment technologies for rural domestic sewage include biological approaches (such as anaerobic–anoxic–oxic, membrane bioreactors, anaerobic digestion, and septic tanks) [75], ecological approaches (such as constructed wetland, ponds, and soil infiltration systems) [76], and combined treatment approaches [77,78]. Zhong et al. used bibliometric software to analyze 512 relevant papers between 1991 and 2022, and concluded that constructed wetlands are the most widely used option in treating rural domestic sewage due to the ease of combination with other treatment technologies [77].

Our studies showed that “anaerobic/aerobic–imitation ecological pond” could be introduced in scattered-living regions where it was difficult to lay out a pipeline network. This technology was demonstrated in Datang Village of Chaohu City, and the removal rates of TN and TP could both reach 60.0% with a processing amount of 180 m^3^/d (3000 residents). For sewage discharged in concentrated areas, the technology of “household sewage–scattered collection–dispersed anaerobic–centralized transport–distribution pond–concentrated multistage land treatment–effluent” was demonstrated in the Dianchang New Village of Chaohu City. The removal rates of COD, TN, and TP could reach 62.5%, 47.9%, and 70.0%, respectively (200 m^3^/d). Moreover, the combination of biological and ecological measures was also feasible, including “anaerobic–anoxic–water dropping–biocontact oxidation–plant filter bed”, according to the sewage emission characteristics and local climate conditions [79,80].

### 3.4. Planning and Construction Technology of Eco-Agricultural Industrial Park

**Figure 1:**
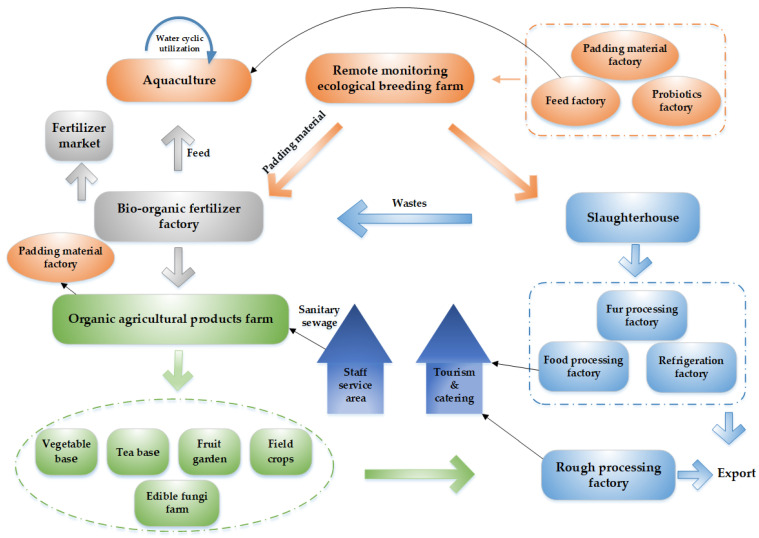
Eco-agricultural park system.

Based on the above key technologies, we put forward the technology system of an eco-agricultural industrial park (Figure 1). Concretely, an eco-agricultural industrial park of 333~667 hm^2^ was constructed with the core of the deep-litter housing system based on the existing farming and breeding industries. In the upstream chains, the micro-ecological industry was vigorously developed to produce green ecological feed, and thus the feed utilization rate was improved and the hazards of organic matter and heavy metals were slowed down. At the same time, multiple sources of agricultural waste (such as straw, sawdust), which were difficult to discard, could be used to produce padding materials of deep litter, and subsequent research on material replacement and optimal allocation schemes could be carried out. In the process of breeding, oral probiotics were added to improve animals’ intestinal micro-ecological environment and create an odorless breeding space. In the downstream industry chain, a marketing network was established, including the slaughterhouse, fur processing factory, and processing industry, with the purpose of improving the additional value of products; meanwhile, the abandoned padding material could be reutilized to produce high-quality biological organic fertilizer, which could be used in the planting base of pollution-free green fruits, vegetables, tea, field crops, and edible fungi, etc. In addition, the circulation industry chain system could be used as a demonstration model to promote the development of agricultural tourism.

Overall, the construction of an ecological industrial park based on the eco-agricultural industrial chain, integrated with a “planting–breeding–agricultural processing–rural living” technology system, could achieve the circulation and extension of the nutrient and material chain and also a win–win scenario of water resource protection and economic benefits. This technology integrates pollution control and resource utilization, which is an effective solution for agricultural NPS pollution control.

## 4. Conclusions and Prospects

Agricultural NPS pollution is the main source of water pollution and has become a bottleneck in the development of modern agriculture in China. An effective method is to control the agricultural NPS pollution through constructing an ecological park with the integration of the eco-agricultural industrial chain.

In the chain of the eco-agricultural industry, suitable technology should be selected according to the local characteristics from different stages of the source, the process, and the end. In detail, the scientific and proper use of pesticides and fertilizers should be promoted, as well as the application of the biological humic acid organic fertilizer in cultivation. In terms of breeding, it is important to control the source of ecological feed to achieve nutrient recycling. In the processing of agricultural products, all related wastes should be fully utilized for ecological feeds and biological organic fertilizers. In the field of rural living, suitable technology should be chosen according to the actual situation in order to achieve in situ sewage disposal and recycling utilization. Finally, based on all these technological measures and helpful policymaking procedures, water resource protection and water quality improvement could be realized ultimately with the combination of these technologies in the chain of the eco-agricultural industry.

## Data Availability

Not applicable.
